# Endogenous Anti-Cancer Candidates in GPCR, ER Stress, and EMT

**DOI:** 10.3390/biomedicines8100402

**Published:** 2020-10-09

**Authors:** Rohit Gundamaraju, Wenying Lu, Iman Azimi, Rajaraman Eri, Sukhwinder Singh Sohal

**Affiliations:** 1 ER Stress & Mucosal Immunology Group, School of Health Sciences, University of Tasmania, Launceston, TAS 7248, Australia; Raj.Eri@utas.edu.au; 2 Respiratory Translational Research Group, Department of Laboratory Medicine, School of Health Sciences, University of Tasmania, Launceston, TAS 7248, Australia; wenying.lu@utas.edu.au (W.L.); sukhwinder.sohal@utas.edu.au (S.S.S.); 3 School of Pharmacy and Pharmacology, College of Health and Medicine, University of Tasmania, Hobart, TAS 7001, Australia; iman.azimi@utas.edu.au

**Keywords:** ER stress, GPCR, LPA, EMT, cancer progression, migration, cancer

## Abstract

The majority of cellular responses to external stimuli are mediated by receptors such as G protein-coupled receptors (GPCRs) and systems including endoplasmic reticulum stress (ER stress). Since GPCR signalling is pivotal in numerous malignancies, they are widely targeted by a number of clinical drugs. Cancer cells often negatively modulate GPCRs in order to survive, proliferate and to disseminate. Similarly, numerous branches of the unfolded protein response (UPR) act as pro-survival mediators and are involved in promoting cancer progression via mechanisms such as epithelial to mesenchymal transition (EMT). However, there are a few proteins among these groups which impede deleterious effects by orchestrating the pro-apoptotic phenomenon and paving a therapeutic pathway. The present review exposes and discusses such critical mechanisms and some of the key processes involved in carcinogenesis.

## 1. Introduction

G protein-coupled receptors (GPCRs) have emerged as key players in tumour growth and metastasis and are regarded as suitable biomarkers for early diagnosis of cancer and in the pharmacological designing of anti-cancer drugs. They are often activated by various factors like chemokines, genetic mediators etc. [1]. Tumour progression is also associated with endoplasmic reticulum (ER) stress. ER stress initiates unfolded protein response (UPR) because of challenging conditions like hypoxia and other environmental perturbations. Activation of the UPR strongly modulates tumour cells’ secretory switch during cancer development [2]. Epithelial to mesenchymal transition (EMT), on the other hand is a long recognised mechanism in epithelial tumours where it aids in increased motility and invasiveness. EMT is ideally initiated by oncogenic pathways modulated by growth factors like Src, Ras, Ets, integrin, Wnt/β-catenin and Notch signalling [3].

GPCRs are a diverse super family of seven transmembrane proteins that comprise one of the largest families in human genome [4]. GPCRs contribute to a number of physiological capabilities during tumorigenesis [5] and are vastly involved in the control of virtually all cell types. Their structure allows for binding of highly diverse ligands, thus they are considered to be the most druggable family of proteins. Loss of balance in the activation of these receptors may result in triggering of conditions such as carcinogenesis. Mechanisms such as GTP hydrolysis, second messenger related protein kinases (e.g., PKA and PKC), G-protein-coupled receptor kinases (GRKs), and arresting prevent the malfunctioning of GPCR signalling. GRKs in general phosphorylate their target GPCR in order to prevent excessive cellular signalling. GRKs are considered to be negative regulators of GPCR activity and are involved in tumorigenesis through processes such a cell death, proliferation, invasion and vascularisation [6]. Nearly 108 GPCR targets are available but fewer than eight are in the anti-cancer class [7]. Moreover, understanding how GRKs regulate GPCR activity may greatly aid in understanding oncogenesis and respected therapeutics.

Activation of GPCR expressed in various cells has been found to stimulate ER stress [8]. Arrestin-1(ARR-1), the GPCR protein in Caenorhabditis elegans, was found to control immune homeostasisby controlling UPR and p38 MAPK signalling pathway [9]. Indeed, ARR-1 was essential for GPCR signalling that controls UPR and various neural pathways associated with ER stress [9]. Furthermore, NPR-1, in a neural circuit setting, controls the p38/PMK-1 MAPK pathway required for innate immunity proposing that GPCRs may engage in neural circuits that receive inputs from either pathogens or infected sites and consolidate them to coordinate appropriate immune responses [10]. Similarly, OCTR-1 also regulates the p38/PMK-1 MAPK pathway and other UPR pathways [11]. GPCRs were reported to be key players not only in cancers but also in inflammation-related diseases such as ulcerative colitis and Crohn’s disease. OGR1 was identified as a classic example of GPCR protein expressed in gut-related inflammatory diseases, where it regulates ER stress through the IRE1α-JNK signalling pathway and blockage of autophagosomal degradation [8]. This body of evidence forms a strong understanding between GPCR and ER stress.

**Figure 1:**
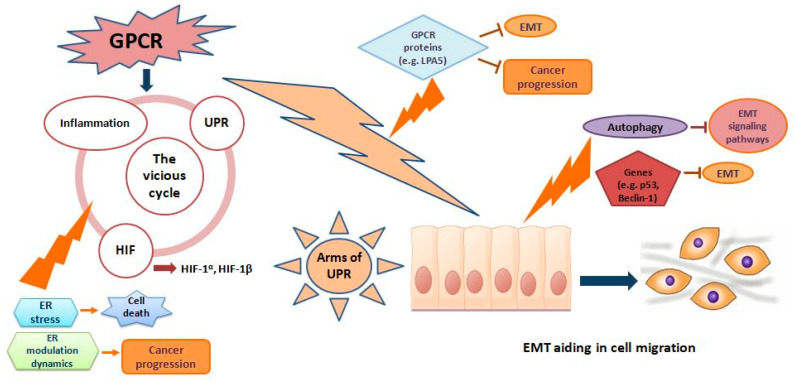
Target sites of the endogenous candidates of G-protein coupled receptors (GPCR), Endoplasmic reticular stress (ER) stress and epithelial mesenchymal transition (EMT). Mounting evidence suggests that GPCR activates unfolded protein response (UPR) in various cancers via mediators like inflammation. Sublethal UPR activation and signalling via IRE1a, PERK, and ATF6a sustain multiple cell-intrinsic and cell-extrinsic mechanisms of tumour progression. ER-stress-mediated activation of central signalling hubs, such as HIF1a, STAT3, NRF2, and NFkB, aides cell survival under harsh microenvironmental conditions and preserves tumour-initiating cell function. Intrinsic cancer cell apoptotic resistance is likely crucial for harnessing ER stress to enhance tumour growth. GPCR protein LPA5 is to exhibit anti-cancer potential by inhibiting mechanisms like EMT. EMT pathways and genes like p53 and Beclin-1 play a positive role in cancer therapeutics. Likewise, certain modulations of ER dynamics and proteins aids in protecting cells from gaining pro survival capability and impeding mechanisms like inflammation.

The course of linkage of GPCR to EMT progression is of interest in cancer therapeutics (Figure 1). EMT is a process whereby epithelial cells lose their apico-basal polarity and strong cell contact, and acquire spindle-like morphology with greater motility [12]. This process is important during physiological phenomena such as embryogenesis and wound healing, as well as pathological events such as cancer metastasis and drug resistance [13,14]. Chemotactic migration is regarded as a key aspect of EMT and cancer progression, has been found to be promoted by the activation of chemotactic GPCRs via impairment of autophagosome biogenesis in U87 glioblastoma cells [15]. In addition, EMT also plays an important role in the development of resistance to EGFR tyrosine kinase inhibitors, such as gefitinib, in non-small-cell lung carcinoma (NSCLC) [16]. Fascinatingly, mesenchymal like cells modulate, detour EGFR signalling and find ways to migrate. Mesenchymal-like NSCLC cells evince aberrant PDGFR and FGFR expression and autocrine signalling through these receptors can activate the MEK-ERK and PI3K pathways.Another GPCR family member, GPR171, identified as a potential tumour-promoting gene, is also overexpressed in lung cancer [17]. Studies have shown that GPR171 enhanced proliferation [17] and metastasis of lung cancer [17,18] in an EGFR-independent manner. The functional crosstalk between GPCRs and EGFR linked to EMT could be a potential target for inhibiting EMT-associated metastasis in lung cancer [19]. The interaction between GPCRs and EGFR also contributes to the progression of other cancers, such as colon, breast, and head and neck tumours [20,21]. In addition, GPCRs are also known to be involved in tumour progression by coupling with the Gs-, Gi- and Gq- protein-signalling pathways. For instance, GPCRs (e.g., GPR78 [22,23]) could interact with Rho GTPase (e.g., RhoA and Rac1) by coupling with the Gαq signalling pathway which is involved in cell migration and invasion [24,25,26].GPR78 knockouts significantly suppressed the cell migration in metastatic lung cancer cell linesbecause it affected the cell motility through the activation of Gαq-RhoA/Rac1 pathway thereby notifying its role in cancer metastasis [23]. In colorectal cancer cells, the activation of RhoA and Rac1 signalling was associated with the mTORC1 and mTORC2 activity in regulating EMT and metastasis [27]. GPCRs knockouts could also potentially suppress EMT-associated metastasis in lung cancer via the Rho GTPase pathway, however further work is needed in this direction.

On the other hand, the orchestrating role of ER stress in EMT initiation has been well established (Figure 1). Hypoxia is a factor driving pro-EMT transcription factors, as well as the activation of ER stress markers both in vivo in rat lungs and in vitroin alveolar epithelial cells (AECs) [28]. The involvement of hypoxia and intracellular calcium in EMT induction of AECs was mainly through the activation of ER stress and hypoxia-induced factor (HIF)-signalling pathways [28]. ER stress induced EMT in AECs through Src-dependent pathways, results in fibroblast accumulation in pulmonary fibrosis [29]. Moreover, enhanced ER stress was responsible for induction of EMT in human lens epithelial cells [30]. ER stress up regulated the EMT markers such as N-cadherin, vimentin, fibronectin and α-SMA [28]. Histone deacetylases (HDACs) participate in the regulation of dynamic equilibrium state of histone or non-histone acetylation/deacetylation. ER stress-inducing agents such as tunicamycin and bleomycin induced ER stress and EMT in lung epithelial cells via the up-regulation of HDACs. Inhibition of HDACs attenuated ER stress and the activation of Smad pathway of EMT induction since tunicamycin and bleomycin, the inducers of ER stress were reported to induce EMT in lung epithelial cells via the upregulation of HDACs [31].

GPCRs, ER stress and EMT play key roles in cancer progression, metastasis, and treatment resistance individually and collectively. In this review, we intend to revisit some of the key candidates in the pathways of GPCRs, ER stress and EMT, which have been found to modulate various mechanisms in cancer progression, possess anti carcinogenic potential and have been tested in disease models. With the best of our knowledge this is the first review to summarize these three systems with regards to the endogenous candidates useful in cancer treatment.

## 2. LPA5 Is a Friend in Need among GPCRs

Lysophosphatidic acid (LPA) vastly dictates embryonic development which is an indirect effector of tumour growth, angiogenesis and metastasis, and a serendipitous finding showed cell migration is impeded by LPA in B16F10 melanoma cells [31]. The expression between lysophosphatidic acid receptor-5 (LPA5) and cancer progression has been the subject of debate. LPA treatment was found to reduce cell survival, which has been proved via LPA5 knockdown. A secondary messenger, cAMP, has also been implicated widely in cell-death decisions, acting as a switch, and LPA5 facilitates cAMP accumulation, thereby reducing cell survival. LPA5-mediated signalling also reduced cell survival in MG-63 osteosarcoma cells. LPA5 combines with Gq and G12/13 which in turn activates Rho-mediated signalling resulting in reduced survival in MG-63 cells [32].

Interestingly, LPA5 mediated the inhibitory effect via rise in cAMP and co-activation of protein kinase A (PKA). LPA5 was importantly considered to be an anti-migratory agent due toits ability to elevate cAMP in both wild type and transfected cells [33]. Similarly, pancreatic cancer cell line PANC-1 was screened for similar effects. LPA5 absence stimulated cell motility, invasiveness and angiogenesis which evidently showed the anti-cancer role of LPA5. Conversely, these effects were reversed by LPA6 knockdown. The role of LPA in the regulation of matrix metalloproteinase (MMP) is well documented. The ATX-LPA-LPA1 signalling axis has been shown to induce MMP-9 expression in hepatocellular carcinoma (HCC). The expression levels of the Mmp-2 gene in MFHL5-2 cells depleted for Lpar5 were significantly higher than those in control MFHGFP cells [34]. LPA5 also reduced the cell motility and MMP-9 activation in fibroblast 3T3 cells [35] and sarcoma cells [36]. 

On the other hand, angiogenesis, which is the process of producing new blood vessels to promote metastasis, is regulated by various factors, including Vascular endothelial growth factor (VEGF). Cell motility activity in endothelial cells is majorly regulated by LPA signalling. Recently a connection was discovered between LPA signalling and VEGF. In an investigation, endothelial cells were cultured with a conditioned medium from neuroblastoma cells expressing individual LPA receptors, and both LPA1 and LPA3 were shown to stimulate the cell motility of endothelial cells, correlating with the expression levels of VEGF genes [37]. A later study found contrasting results where LPA5 decreased VEGF expression and negatively regulated cell motility [34]. This theory not only explains the protective nature of LPA5 in carcinogenesis but also the role of LPA in angiogenesis signalling establishing a chemotherapeutic target in sarcomas. 

Autotoxin is an ectonucleotide pyrophosphatase/phosphodiesterase (ENPP) family member which produces LPA. Up-regulated in most of the metastatic cancers, autotoxin was also associated with invasiveness and aggressive metastatic potential of cancers and was positively correlated to tumour angiogenesis in colorectal cancer [38]. Furthermore, evidence has identified autotoxins as secretory proteins in human melanoma cells, and the overexpression of autotoxins was co-relatable to increased motility and invasiveness [39]. LPA and autotoxin expression differ vastly regarding in vitro and in vivo settings. Autotoxin was found to be involved in facilitating B16F10 metastasis in C57BL/6 mice [40,41]. However, under in vitro conditions, there was a diminished cell invasion in similar cells under the influence of LPA [42]. This finding further indicates that negative effects of LPA5 against cell proliferation and migration, which also have been shown in melanoma and pancreatic cancer cells [36], and the exogenous expression of LPA5 in intestinal epithelial cells MSIE lessened cell proliferation. Such evidence greatly supports the role of LPA5 in cancer therapeutics. 

Cancer immune editing is a process adapted by cancer cells in order to evade cell death and reside in harsh environments. Some of the mechanisms employed by the tumours in order to escape harm include CD8T cell response. The CD8T activation by tumour antigen is initiated through T cell antigen receptor (TCR) signalling. Previous deleterious effects of LPA on migration, metastasis and therapeutic resistance were underscored in a study by Oda et al., where LPA5 inhibits CD8 T cell receptor signalling, activation and proliferation [43]. This study not only identified the requirement of LPA5 for negative regulation of TCR-induced calcium mobilisation but also in attenuating antigen-mediated proliferation in vivo. 

## 3. ER Stress: A Friend Apart from Foe?

Endoplasmic reticulum (ER) strives for homeostasis. When ER function becomes overwhelmed with an excessive accumulation of misfolded proteins within the lumen, ER stress is triggered.ER stress releases coping mechanisms to reduce the damage. The adaptation to a stress environment is achieved by ER stress. If the recovery of cellular adaptation fails, long-standing ER stress triggers programmed cell death or apoptosis. On the other hand, growing evidence suggests a novel pathway which helps cells to survive extreme environmental conditions and escape cell death is via up-regulation of ER adaptive measures. The million-dollar question here is whether ER stress is a survivor or a killer [44]. 

Cancer stem cells (CSC) are more resistant towards chemotherapy, consequently sensitising these cells to chemotherapy is a means by which to render them prone to cell death. CSCs treated with salubrinal, a specific inhibitor of eukaryotic translation initiation factor 2a (eIF2α) phosphatase, followed by conventional chemotherapeutic agents resulted in sensitisation of CSC towards oxaliplatin and 5-FU. A similar pattern was observed duringin vivo experiments. Mice treated with salubrinalled to transient UPR activation which increased growth of xenografts derived from colon-CSCs, however combinational treatment with chemotherapeutic agents suppressed the growth of the xenografts, indicating the positive effect of UPR in vitro and in vivo [45]. 

Conditions such as hypoxia are essential for tumour survival. Several cancers up-regulate GRP78 and XBP1 splicing during hypoxia. In colon cancer, hypoxia induces PERK-dependent phosphorylation of eukaryotic translation initiation factor 2a (eIF2a) and translation of ATF4. UPR is vital for tumour growth under hypoxia. PERK inactivation, due to the generation of mutations in its kinase domain, impairs cell survival under extreme hypoxia, and PERK promotes cancer cell proliferation by limiting oxidative DNA damage through ATF4 [46]. 

On the other hand, the same ER stress is useful in promoting cell death. Tolfenamic acid promotes ER stress, resulting in activation of the UPR-signalling pathway, of which PERK-mediated phosphorylation of eIF2a induces the repression of cyclin D1 translation. Moreover, the PERK-eIF2a-ATF4 branch of the UPR pathway plays a role in tolfenamic acid-induced apoptosis in colorectal cancer cells, as silencing ATF4 attenuates tolfenamic acid-induced apoptosis [46].

Plant metabolites have been a promising source of cancer therapeutics for many decades. Esculetin, a coumarin derivative, has been examined on colon cancer cells for its potential anti-cancer activity via ER stress-mediated cell death. Esculetin-induced cell death via the ER stress-mediated pathway increased mitochondrial Ca^2+^ overload and also escalated the level of ER stress response proteins. It was also proven that the cell death was induced by a mechanism of UPR where CHOP is up-regulated and caspase-12 is cleaved. This CHOP initiation mechanism hinders BCL2 family proteins and activates BAK and BAX, thereby inducing apoptosis [47].

Activation of caspase-3, which is an intrinsic cell death pathway, is another mode of inducing apoptosis. Andrographolide treatment leads to apoptosis in numerous cancer cells. One of the pathways that has been elucidated and which has most potential is ER stress-mediated cancer cell death. Upon treatment, there was a significant increase in IRE1-α and spliced XBP-1 which triggers apoptosis. Surprisingly, the Andrographolide-mediated cell death was also dependent upon ER stress given that Andrographolide up-regulated the expression of BAX and also major ER stress markers [48].

Triggering a transcription of heat shock proteins can lead to ER stress-mediated cell death. At the same time, proteasome inhibitors were reported to initiate apoptosis in cancer cells, and functional or mutational changes in some of the ER genes have been associated with malfunctions. XBP1 mutation has been reported in rare myeloma and may be associated with resistance to proteasome inhibitors [49]. Hence, it is evident that this inhibition might play a key role in cancer therapeutics. For instance, PS-341, a di-peptidyl boronic acid derivative, has shown impressive binding to 26 proteasome and has induced cell death in numerous cancer cell lines. With the ability to target 26S proteasome, PS-341 has been logically associated with targeting NF-kB. Traditionally, NF-kB was linked to chemotherapy resistance and PS-341 was tested in order to check its ability in inhibiting NF-kB via chemotherapy-mediated cell death [50]. Surprisingly, it was found that PS-341 could induce topoisomerase-1 inhibitor-mediated apoptosis. The potency of PS-341 was assessed in head and neck SCC (HNSCC) cells [51,52,53]. PS-341 not only lessened NF-kB but also induced cell death via the ER stress pathway. As part of the involvement of ER stress-modulated cell death in the PS-341 mechanism, caspase-4 played a crucial role. A study by Fribley and Wang described the potential mediatory agents in ER stress-mediated cell death involving PS-341. Their review found that the two major mechanisms involved are BH3-only members of the Bcl2family interfering with the cytochrome-c release and via induction of BH3-only proteins Bik and Bim [54]. 

Aspects that need to be emphasized regarding the induction of apoptosis in various tumours include co-expressions or loss of genes such asp53and the time period of ER stress induction. Conceptually, targeting UPR might block ER stress-induced apoptosis and unwittingly promote carcinogenesis. There are few reports of UPR being involved in promoting cancer. Such mechanisms might depend on the intensity and time of the ER stress which we recently proved via dynamics of ER stress [55], where we identified the maximum deleterious effect of ER stress at the 6th hour of the dynamics period in vitro. We even employed unconjugated bilirubin to impede ER stress-mediated cancer progression in LS174T cells via similar time point [56]. This clearly indicates that the exposure or the ER stress mediation in induction of cell death is critical. 

## 4. Good Guys in the EMT Pathway

EMT is known to be an important factor associated with cancers’ progression, metastasis and treatment resistance [57]. Polarised epithelial cells lose their cell-cell adhesion and apical-basal polarity, andgain migratory potential as mesenchymal cells [58]. The transition process involves numerous pathological changes. The cells gradually loose epithelial cell-cell junctional proteins, such as E-cadherin, ZO-1 andcytokeratins, and gain mesenchymal proteins, such as N-cadherin, fibronectin and vimentin [59,60]. EMT is a highly plastic process. Indeed, mesenchymal cells can revert to an epithelial state in a process called mesenchymal to epithelial transition (MET), a process that is critical during the clonal expansion of metastasised cells [12].

There are several signalling pathways driving EMT, including inflammation, transforming growth factor beta (TGF-β), Wnts, NF-κB and Notch pathways [59]. The TGF-β pathway, as a primary inducer of EMT, is activated by binding TGF-β ligands to their cognate TGF-β receptors. With the active TGF-β receptors, TGF-βsignalling complies with Smad2 and Smad3 to lead to EMT [61]. In addition, TGF-βsignalling can also stimulate GTPases, PI3K and MAPK pathways to induce EMT progression [62]. Other signalling pathways, such as Wnt, Notch, AKT-mTOR and NF-κB pathways, induce EMT by activating EMT transcription factors (EMT-TFs) [59]. There are certain transcription factors described as major regulators of EMT, such as Snail, Twist, β-catenin, ZEB1 and ZEB2. EMT-TFs suppress the expression of epithelial proteins [63]. For instance, Snail inhibits the expression of the key epithelial protein E-cadherin, therefore epithelial cells lose the cell-cell junction formation, leading to mesenchymal transition [64]. The EMT process in normal tissues is managed through a complicated regulation of EMT-TFs, with applied regulatory networks operating at different transcriptional and post-translational levels, such as alternative splicing, non-coding RNAs, epigenetic regulatory mechanisms and protein stability [65]. Studies have shown that the role of EMT-TFs in cancer progression is not only to regulate the invasion and dissemination of cancer cells, canbecome a target of interest for anti-cancer therapy [66]. 

**Figure 2:**
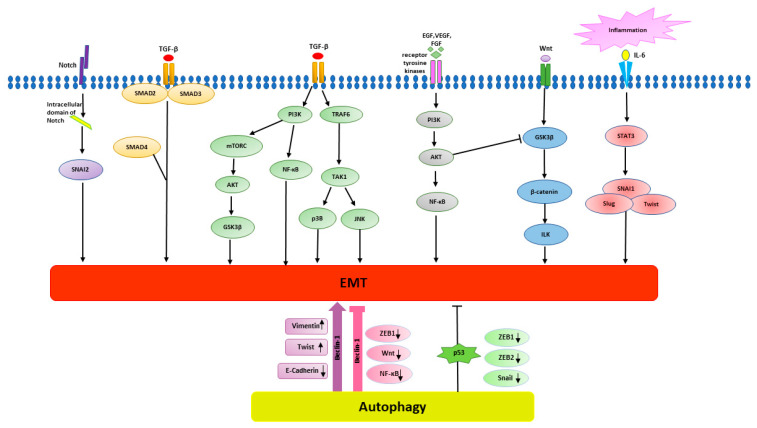
Cross-link between EMT-induced signalling pathways and Autophagy-inducedsignalling pathways. EMT-mediated signalling pathway includes Notch, TGF-β, receptor tyrosine kinases, Wnt, and inflammatory pathways. TGF- β signalling complies with SMAD2 and SMAD3to lead to EMT. TGF- β signalling also activates PI3K-mTOR-AKT-GSK3b pathway to induce EMT. Wnt signalling drives EMT through GSK3β inhibition and β-catenin stabilization. Wnt, Notch and inflammatory pathways induce EMT by activation of EMT transcription factors, including SNAI1, β-catenin, Twist and Slug. Receptor tyrosine kinases activate PI3K-ATK pathway via activation of growth factors, such as EGF, VEGF and FGF. Autophagy triggers EMT by up-regulating mesenchymal markers including vimentin and Twist and down regulating epithelial marker E-cadherin through Beclin-1 pathway. Beclin-1 pathway can activate autophagy to suppress EMT via down-regulating EMT transcription factors, such as ZEB1 and inhibiting Wnt and NF-κB pathway. P53 inhibits EMT by decreasing the expression of EMT transcription factors, such as ZEB1/ZEB2, Snail.

Autophagy is another principal biological process involved in the development of cancer, and there is a complex link between autophagy-corresponding and EMT-corresponding signalling pathways (Figure 2). Studies have shown that EMT signalling pathways can trigger or inhibit autophagy. As well as being associated with the initiation and suppression of EMT, autophagy also supports EMT in the viability of potentially metastasis of cancer cells [67]. For instance, autophagy deterioration was demonstrated by suppressing autophagy-related genes 5 (ATG5), ATG7 or Beclin-1, resulting in an increase of cell motility and invasiveness with the up-regulation of Snail and Slug, two of the major EMT-TFs [68]. On the other hand, autophagy prevents EMT, and the autophagy activation may decrease the gaining of the EMT phenotype in cancer cells. Autophagy is regulated by PI3K/AKT/mTOR, Beclin-1, p53 and JAK/STAT signalling pathways, which have a dramatic impact on the EMT process [66]. EMT-correlated signalling pathways, such as integrin, Wnt, NF-κB, and TGF-β signalling pathways, also play an essential role in autophagy [66]. 

Beclin-1 activates autophagy and accelerates EMT by up-regulating vimentin and Twist expression and decreasing E-cadherin expression [69]. In contrast, Beclin-1 activated autophagy down-regulates MMPs’ expression to inhibit EMT and also inhibit EMT via down-regulating ZEB1, Wnt1 and NF-κB. NF-κB activation is associated with aggressiveness and the metastatic potential of carcinomas [70]. The NF-κB pathway promotes EMT by up-regulating related EMT markers, including Snail1, Slug and Twist1 [71], and inhibits autophagy by down-regulating the Beclin-1 pathway [66]. In addition, the study also indicated that Beclin-1 gene knockout may promote EMT and carcinogenesis by activating the Wnt1 and NF-κB pathways resulting in cancer cell metastasis. However, knockdown of Beclin-1 via small interfering RNA (siRNA) suppressed the autophagy activation, consequentially suppressing EMT and the invasiveness of colon cancer cells through cooperating down-regulation of vimentin and Twist and up-regulation of E-cadherin [72]. This result suggests that inhibiting Beclin-1 induced autophagy would be an effective anti-cancer strategy.

P53 is an important suppressor protein of cancer. P53 mediates cancer inhibition by down-regulating autophagy-correspond signalling pathways PI3K/AKT/mTOR via interaction with PTEN, which furthers the up-regulation of autophagy [66]. P53 can also mediate cancer suppression by regulating EMT inhibition through decreasing the expression of EMT-TFs, including ZEB1, ZEB2 and Snail, via activation of the relevant micro RNA of EMT inhibition [73,74]. Interestingly, mutant p53 can promote EMT and mitochondrial fission that in turn promotes autophagy [75].

Autophagy and EMT both play an important role in the biological processes of induction and development of cancer. Understanding the complicated link between autophagy and EMT is necessary for designing a cancer therapy strategy. Autophagy activation not only supports the cells’ survival during the EMT, but also functions as the tumour-suppressive signal, which inhibits the early phase of metastasis and activation of the EMT. Hence, regulating EMT by targeting autophagy is a promising potential strategy for cancer therapy. Currently, translational applications of autophagy activators such as rapamycin, and autophagy inhibitors such as chloroquine and 3-methyladenine to regulate the EMT process, have been utilised in anti-cancer therapy [67,76,77]. 

Table 1 shows the endogenous candidates which play an active role in cancer therapeutics. 

Table 2 enlists the various drugs and metabolites with essential anti-cancer activities in their respective systems which are widely useful for cancer therapeutics.

## 5. Conclusions

Some of the malfunctions in GPCR genes are associated with misfolding mutant receptors in the ER. Understanding the GPCR-mediated mechanisms in cancer, identifying possible role of ER stress in hampering of pro-survival mechanisms would be extremely important. These hallmarks of events regulate crucial mechanisms such as EMT which is the most pivotal step in metastasis. Hence, a proper elucidation of these candidates would help in identifying potent molecular targets for regulation or modulation of tumour progression in numerous cancers. 

## Figures and Tables

**Table 1:** Endogenous anti-cancer agents.

Endogenous Candidates	System	Action	Outcome or Drug Development	References
Beclin-1	EMT	Activates autophagy, down regulates MMPs expression to reduce EMT.	Pathway for novel drug discovery	[68,69]
P53	EMT	Down regulates PI3K/AKT/mTOR via interaction with PTEN	Pathway for novel drug discovery	[73]
LPA5	GPCR	Aids in inhibiting carcinogenesis and attenuating cell migration and proliferation	Essential pathway in drug discovery	[42,43]

EMT-epithelial mesenchymal transition; GPCR-G-protein coupled receptor.

**Table 2:** Anti-cancer agents developed from ERS and EMT.

Drugs	System	Action	Outcome or Drug Development	References
Salubrinal	ERS	eIF2α inhibitor for chemo sensitising CSC.	Useful in chemotherapy	[43]
Tolfenamic acid	ERS	PERK-mediated phosphorylation of eIF2a	Anti-cancer drugs	[44]
Esculetin	ERS	Increases mitochondrial Ca2+overload and inducing ERS mediated cell death of cancer cells.	Anti-cancer drugs	[45]
Andrographolide	ERS	Increase in IRE1-α and spliced XBP-1 leading to ERS mediated cell death	Anti-cancer drugs	[46]
PS-341	ERS	Topoisomerase-1 inhibitor-mediated apoptosis.	Anti-cancer drugs	[52]
Chloroquine	EMT	Autophagy inhibitors	Anti-cancer drugs	[76,77]
3-methyladenine	EMT	Autophagy inhibitors	Anti-cancer drugs	[76,77]

ERS-Endoplasmic reticular stress.

## Abbreviations

GPCR	G protein coupled receptors
EMT	Epithelial mesenchymal transition
EGFR	Epidermal growth factor receptor
LPA	Lysophosphatidic acid
ERS	Endoplasmic reticular stress
UPR	Unfolded protein response
GRK	G protein coupled receptor kinase
ARR	Arabidopsis type B cytokinin
NSCLC	Non small cell lung carcinoma
ENPP	Ectonucleotide pyrophosphatase/phosphodiesterase
MAPK	Mitogen activated protein kinase
AEC	Alveolar epithelial cells
HDAC	Histone deacetylases
STAT	Signal transducer and activator of transcription proteins
NRF	Nuclear receptor factor
MMP	Matrix metallopeptidases
HCC	Hepatocellular carcinoma
VEGF	Vascular endothelial growth factor
CSC	Cancer stem cells
GRP78	78-kDa glucose-regulated protein
XBP1	X-Box Binding Protein 1
CHOP	C/EBP homologousprotein
TGF	Transforming growth factor
PTEN	Phosphatase and tensin homolog
FGF	Fibroblast growth factor
EGF	Epidermal growth factor
PKA	Protein kinase
PDGF	Platelet derived growth factor
EMT-TFs	EMT transcription factors
PERK	PKR-like ER kinase
Bax	Bcl-2 associated X protein
HNSCC	Head and neck squamous cell carcinoma
Zeb 1	Zinc finger E-box-binding homeobox 1
